# Sunlight perception and outdoor thermal comfort in college campuses: a new perspective

**DOI:** 10.1038/s41598-023-43077-y

**Published:** 2023-09-26

**Authors:** Shaobo Ning, Wenqiang Jing, Zhemin Ge

**Affiliations:** 1 Department of Architecture, School of Human Settlements and Civil Engineering, Xi’an Jiaotong University, Xi’an, 710049 China; 2 School of Human Settlements and Civil Engineering, Xi’an Eurasia University, Xi’an, 710055 China; 3 Engineering Research Center of Urban Intelligent Construction, Universities of Shaanxi Province, Xi’an, China

**Keywords:** Environmental sciences, Environmental impact

## Abstract

The thermal comfort of outdoor spaces in colleges and universities is crucial for promoting outdoor activities and relieving psychological pressure. To evaluate outdoor thermal comfort from a new perspective, this study investigated subjects' sunlight perception through physical measurements and questionnaires. Sunlight perception was delineated through a combination of subjective assessments and objective measurements. Subjective assessments encapsulated thermal comfort and sensation votes, and sunlight sensitivity. Objective measurements incorporated physical environmental data such as temperature, humidity, wind speed, illumination, and solar radiation. The Universal Thermal Climate Index (UTCI) was used to examine the thermal sensation of subjects under different sun perceptions to reveal the effect of sunshine sensitivity on subjects. The results showed that in terms of subjective perception, the proportion of people who felt hot outdoors increased with the increase in sunlight perception. Additionally, with the change of sunlight perception, the expected temperature of the crowd also changed. As the sunlight perception changed from weak to strong, the desired temperature of the winter population changed from 21.2 °C to 17.7 °C, and the desired temperature of the autumn population changed from 23.8 °C to 19.8 °C. Appropriately increasing shade outdoors in autumn would enhance the comfort of the crowd, while appropriately increasing the light place in the winter outdoors would enhance the comfort of the crowd. These findings provide valuable insights for thermal comfort design and future research in colleges located in cold areas.

## Introduction

Currently, in the planning and design of college campuses, the focus is often on the spatial layout and architectural form of the campus buildings and landscape as a whole^1^. Insufficient attention is paid to the value of outdoor space and the importance of environmental comfort^1^. However, as a vital location for relaxation, socialization, and entertainment for teachers and students, the quality and comfort of outdoor space significantly impact the frequency and possibility of its use^2^, as well as the cultural atmosphere and vitality of the campus, and the development of students' personality and communication skills. Furthermore, with the expansion of colleges and universities, dormitory buildings, teaching buildings, and other facilities have increased building density and personnel density, causing issues such as poor ventilation and lighting in the physical environment, resulting in physiological discomfort. In this context, having a good and comfortable outdoor space is particularly important as it can alleviate and improve physical and psychological discomfort. Therefore, the study of outdoor activity space comfort on campus is of great social application value and significance.

As early as the 1940s, some scholars began to explore the relationship between the outdoor building environment and thermal comfort. The American Society of Heating, Refrigeration and Air Conditioning Engineers (ASHRAE) identified external environmental factors and physiological parameters as the primary factors that affect human thermal comfort. External environmental factors include outdoor temperature (T), relative humidity (RH), wind speed (V), and mean radiation temperature (Tmrt), while physiological parameters include clothing volume (Clo) and metabolic rate (Met)^2^. However, there are significant gaps in the applicable scope of the current comfort index for outdoor unstable conditions, particularly in cold climates where few studies have been conducted^3^.

The thermal comfort index can be divided into two categories: empirical indices and mechanism indices^3^. Urban environmental scholars proposed various outdoor thermal comfort mechanism indicators to establish the corresponding relationship between the outdoor environment and human thermal sensation and identify improvement measures^4^. Mechanism indicators are based on the thermodynamic equation and primarily include Heat-stress Index (HIS), Effective Temperature index (ET*), Standard Effective Temperature (SET*), Outdoor Standard Effective Temperature (OUT-SET*), Predictive Mean Vote (PMV), Physiological Equivalent Temperature (PET), Universal Thermal Climate Index (UTCI)^5^, etc. These indices are mostly based on outdoor human thermal balance models, which are widely used in current studies. Fanger et al.^6^ proposed the steady-state equation of human energy, which used PMV as an evaluation index. The two-node model proposed by Gonzalez et al.^7^ used new Effective Temperature (ET*) and Standard Effective Temperature (SET*) as evaluation indices. The MEMI model proposed by Mayer et al.^8^ expanded the two-node model and used physiological equivalent temperature (PET) as the indicator model. Among the current thermal comfort indices, PET^9^ and UTCI^10^ are the main indices applicable to outdoor thermal environment evaluation. There are also some indices that have not been extensively demonstrated, such as ETVO^11^, ETU^12^, ETF^13^, COMFA^14^, etc. Thermal comfort evaluation indices applicable to cold regions mainly include Predictive Mean Vote (PMV), Physiological Equivalent Temperature (PET), and Universal Thermal Climate Index (UTCI)^15^. The PMV index is directly based on the actual temperature for questionnaire voting and intuitively reflects the impact of temperature on human comfort through statistical data. However, the actual comfort level of the human body is influenced by various micro-climate factors and cannot be measured by a single index. PET considers human comfort and is suitable for various climates, but requires more test parameters^16^. UTCI expresses the equivalent temperature of the reference environment^17^ and is applicable to climatic conditions at different scales^18^. Overall, UTCI is more suitable for calculating thermal comfort in outdoor campus spaces in cold areas.

The focus of outdoor thermal comfort research is the microclimate of outdoor spaces. For instance, a study encompassing 14 urban spaces across five European cities demonstrated a strong correlation between microclimate, particularly air temperature and solar radiation, and human comfort^19^. This finding is echoed in a study conducted in Lisbon, Portugal, which found that residents' air temperature preferences were seasonally dependent and significantly influenced by wind speed and sunlight perception^20^.

Further evidence of seasonal variation in thermal comfort comes from a study in Tianjin, where Lai et al.^21^ found that residents' thermal sensations varied seasonally, with air temperature and sunlight perception being the most influential factors. Similarly, Lin et al.^22^ reported that air temperature and average radiation temperature had the greatest impact on human thermal sensation, followed by sunlight perception and wind speed. Importantly, they found a strong positive correlation (0.65) between sunlight perception and thermal comfort ratings.

These studies collectively underscore that people's outdoor thermal comfort is not static but varies seasonally and under different sunlight conditions. However, while these studies provide valuable insights, they do not fully explore the subjective perception of sunlight and its impact on thermal comfort in outdoor campus spaces in cold regions. This is the gap our study aims to fill.

The aforementioned scholars' research demonstrates that outdoor thermal comfort for people is not only strongly correlated with temperature but also dependent on sunshine sensitivity. Nevertheless, there is a lack of research regarding the influence of different sunlight perceptions on the thermal comfort of people on campuses in cold areas. Sunlight perception is the subjective feeling of outdoor people, which is used to determine the feeling of outdoor people about the intensity of outdoor sunlight perception refers to individuals' subjective feelings about the intensity of outdoor sunlight. A subjective questionnaire is used for data collection, similar to thermal sensation vote (TSV), thermal comfort vote (TCV).This study introduces a novel methodology for categorizing outdoor populations on campuses based on varying sunshine sensitivity intensities. While prior research has underscored the significance of sunshine sensitivity in evaluating outdoor thermal comfort, our approach delves deeper. We not only recognize sunshine sensitivity as a pivotal factor but also empirically demonstrate its direct impact on neutral and expected temperatures, especially in winter scenarios. By conducting a comparative analysis among college students in Xi'an, we aim to shed light on how sunshine sensitivity nuances can influence outdoor thermal comfort. This study aims to investigate two main areas:

1. To investigate the differences in subjective perceptions of thermal sensitivity among people with different perceptions of sunlight in outdoor campus spaces in cold regions.
2. To investigate the differences in subjective perceptions of thermal comfort among people with different perceptions of sunlight in outdoor campus spaces in cold regions.

Based on these objectives, we propose the following hypotheses:

H1: There are significant differences in subjective perceptions of heat perception among individuals exposed to different levels of sunlight in outdoor campus spaces in cold regions.

H2: There are significant subjective perceptual differences in thermal comfort among individuals exposed to different levels of sunlight in an outdoor campus space in a cold region.

These hypotheses will be tested by a comprehensive survey and physical measurements of the environment.

## Research method

### Research location

**Figure 1:**
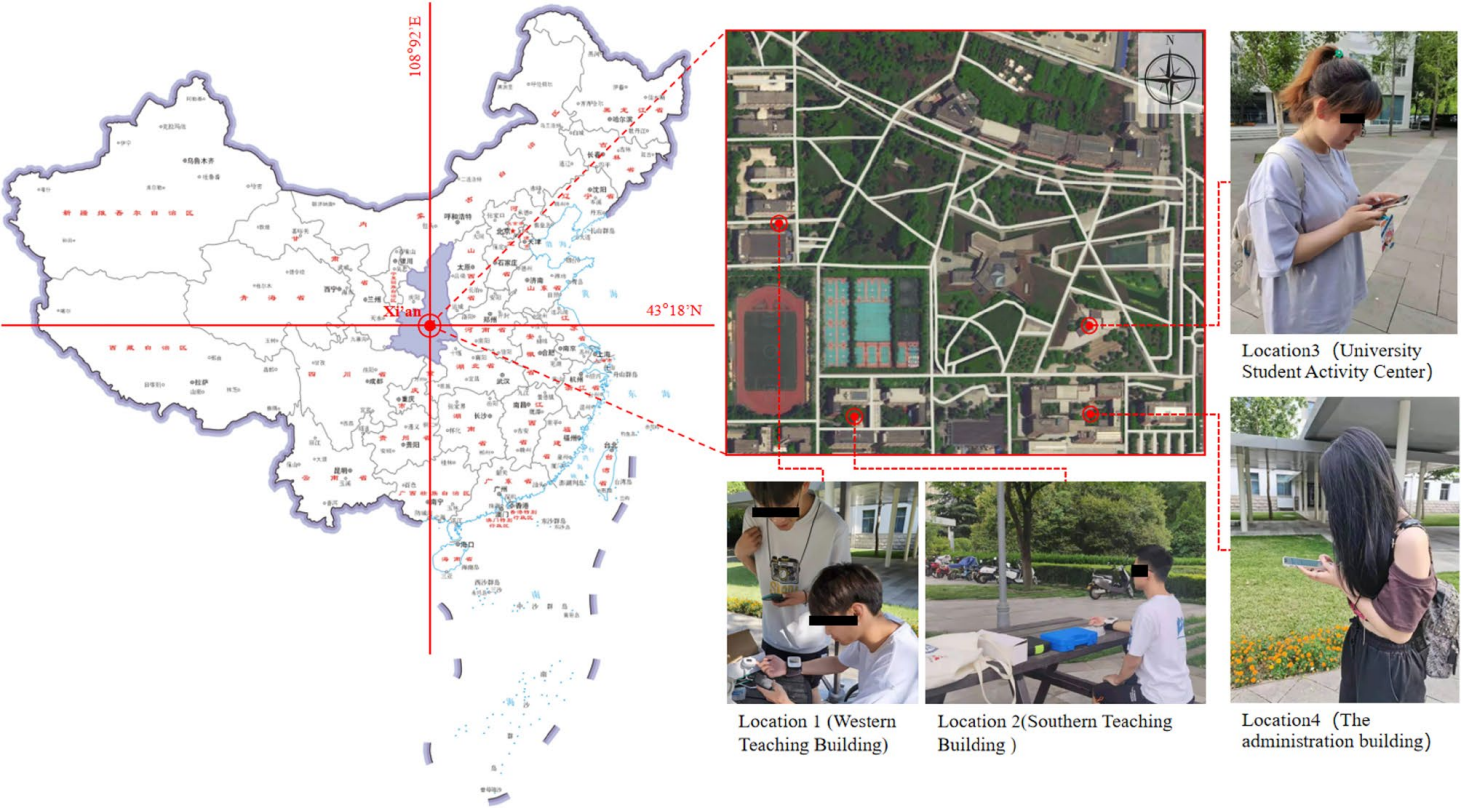
Research location. Source: PPTX (2016). Retrieved from[http://m.qpic.cn/psc?/V53NlG4e4RfKL62K0hiA1W2rRr432PG9/ruAMsa53pVQWN7FLK88i5shCFRJUR3zYZGhkhafkmyeQaMFMbkr.CpeiZl8UjFk9WDyrzKeLrvVA0ddaF6ScdawuD02l6XP7w*46SV8GRnQ!/b&bo=hgclBAAAAAADB4I!&rf=viewer_4].

Xi'an (107.40 degrees to 109.49 degrees East, 33.42 degrees to 34.45 degrees North) is a cold region, and the Xi'an area is characterized by cold and dry winters and hot and humid summers, with 2200 to 3000 h of sunshine and 502 to 586 kJ/cm^2^.year of radiation throughout the year. The location of this research is Xi'an Eurasia University. This research was conducted at Xi'an Eurasia University, an outdoor public space frequently used by students. In this study, the outdoor public space of Xi'an Eurasia University was selected. The criteria for the site selected include the following considerations. Firstly, the chosen site is an outdoor space where students often play. Secondly, the location chosen needs to include both places that are directly exposed to sunlight and places that are not directly exposed to sunlight. According to the above two criteria, we selected four outdoor spaces of Xi'an Eurasia College, as shown in Fig. 1. Location 1 has an awning, so it is a location that is not directly exposed to sunlight. Location 2 has tall trees that provide shade, so it is a location that is not directly exposed to sunlight. Locations 3 and 4 do not have shade and are therefore locations with direct exposure to sunlight. Location 1, equipped with an awning, is shielded from direct sunlight. Location 2, shaded by tall trees, also avoids direct sunlight. In contrast, Locations 3 and 4 lack shade, resulting in direct sunshine sensitivity.

### Questionnaire

The questionnaire was divided into two parts: (1) subjective perceptions of the respondents. Respondents' subjective thermal comfort value thermal comfort vote (TCV), thermal sensory value thermal sensation vote (TSV), heat acceptance and sunlight sensitivity of the current environment (Table 1). (2) Personal information of respondents, which provides basic information about the respondent, such as age, sex, health status, etc. The gender distribution in the sample was balanced, with slightly more males than females, but the difference was not statistically significant. According to the actual situation, investigators record the survey location, survey time, the movement state and clothing of the respondents, and record the physical information of the current environment, such as temperature, humidity, wind speed, illumination and solar radiation, according to the measuring instrument.

**Table 1:** Subjective evaluations used in the questionnaire.

Scores	TCV	TSV	Psychological expectations of ambient temperature	Sunshine sensitivity
3	Very comfortable	Hot	–	–
2	Comfortable	Warm	–	–
1	A little bit comfortable	Slightly Warm	Prefer cooler conditions	Strong
0	Medium	Neutral	Constant	Moderate
− 1	A little bit uncomfortable	Slightly Cool	Prefer warmer condition	Not strong
− 2	Uncomfortable	Cool	–	
− 3	Very uncomfortable	Cold	–	–

### Outdoor climate

Field surveys were conducted at four sites of Xi’an Eurasia College. In addition to randomly selecting respondents for the subjective evaluation survey, the investigators also measured and recorded physical environmental factors, such as air temperature, relative humidity, wind speed, and solar radiation, so that subjective evaluations correspond to objective measurements, making research results more reliable. The specific measuring instrument model, measuring range, measuring accuracy and use are shown in Table 2. Temperature, relative humidity and wind speed are unstable to a certain extent. When measuring, pay attention to the fluctuation of the measured value and whether there is an extreme value to make the measurement result is more objective and stable.

**Table 2:** Basic parameters of measuring instruments used.

Instrument model	Measured parameters	Measurement range	Measuring accuracy
Anemometer (wind0501)	Wind speed	0.2–10 m/s	± 0.02 m/s
Multifunctional detector (xjbthi210501)	Temperature	− 40 °C ~ + 125 °C	± 0.3 °C
	Humidity	0%RH ~ 100%RH	± 2%RH
Bolometer (JT2020)	Global radiation	0 ~ 2KW/m^2^	± 5%W/m^2^

**Figure 2:**
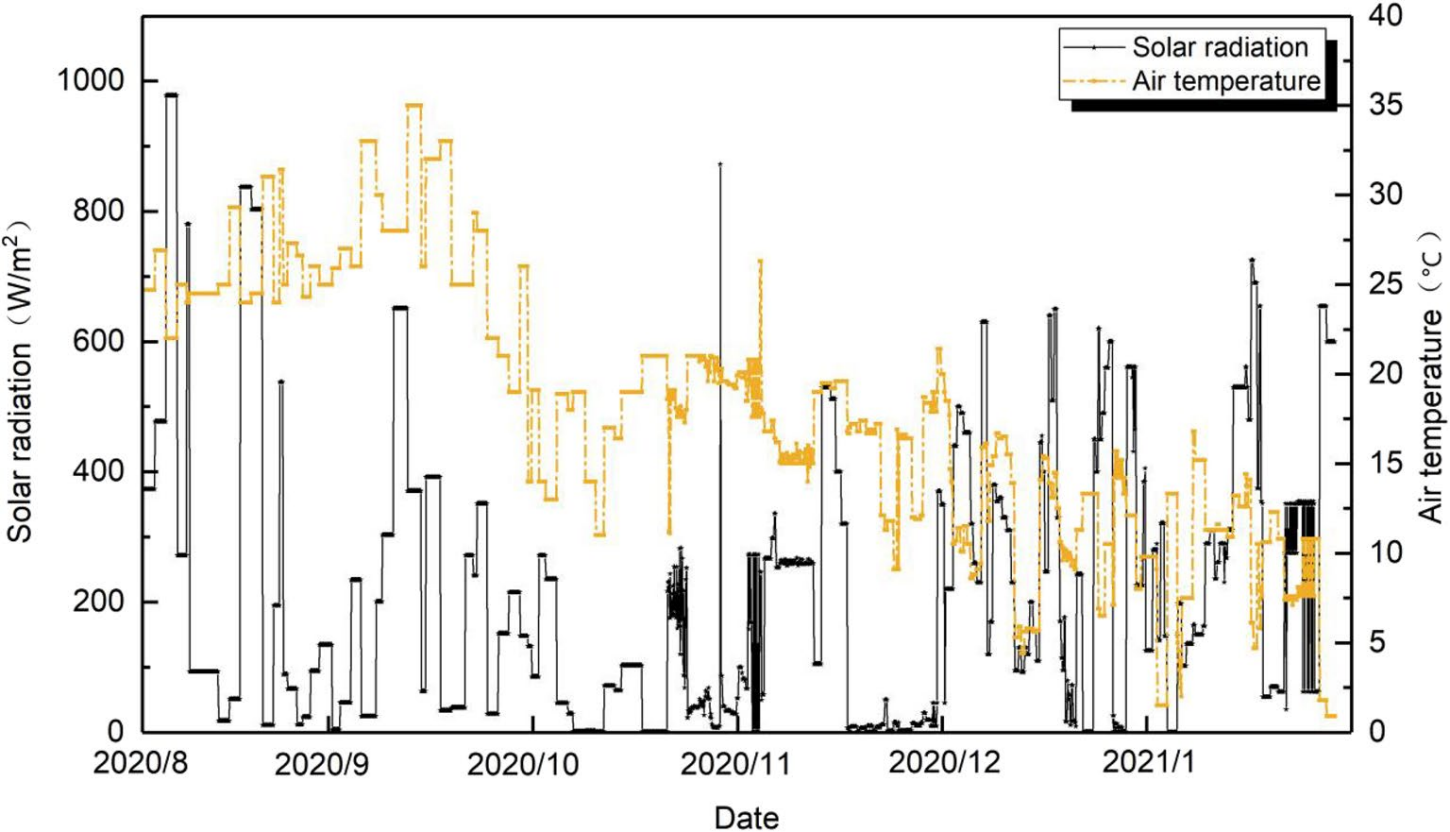
Variation of outdoor solar radiation and air temperature during the survey days.

**Figure 3:**
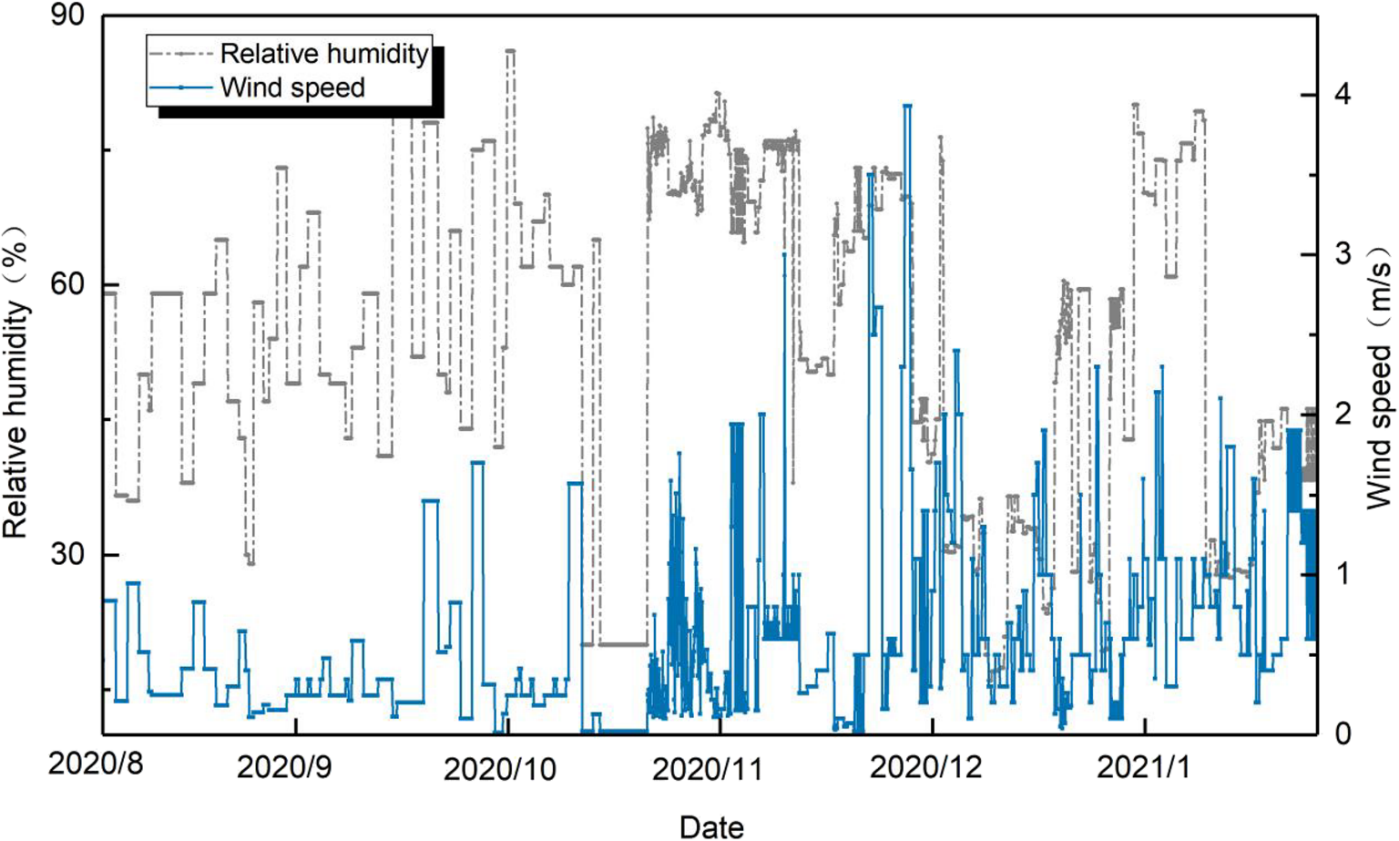
Variation of outdoor relative humidity and wind speed during the survey day.

Figures 2, 3 and Table 3 show the micrometeorological parameters of the survey site during the survey period (August 2, 2020, to January 25, 2021).

**Table 3:** Outdoor weather parameters.

Climate parameters	Maximum value	Average value	Minimum value
Outdoor temperature (°C)	35	18.1	0.9
Relative humidity (%)	86	53.5	16
Wind speed (m/s)	3	0.64	0.02
Solar radiation (W/m^2^)	978	211.9	0.1

### Subjects

A field survey was conducted from August 2020 to January 2021.During trails, four monitoring stations were set up to record meteorological data simultaneously at each space.All instruments were installed 1.5 m above the ground. Since residents attended outdoor activities for a shorter period in winter than that in autumn, trials were run from 9:30 to 16:00 in winter and from 9:00 to 17:00 in autumn. Measurements were performed under conditions without wind, rain or falling snow.

A total of 24 volunteers (14 males and 10 females, of similar age, from 20 to 22 years old) took part in the study (12 participants per day). Clothing insulation and activities of volunteers were determined using ASHRAE standard 55 (ASHRAE, 2005). During the survey, volunteers could choose their clothes freely, and they indicated this choice in the questionnaire. Whenever subjects were subjected to subjective questionnaires. At predetermined intervals, participants filled out the questionnaires. The meteorological parameters at this time were recorded by the investigator. A total of 2395 valid questionnaires were collected, including 1024 in autumn and 1371 in winter. As the research site is a university campus, the respondents are mainly students. The respondents were aged between 18 and 20. There were more males than females, but the difference between males and females was not significant. All respondents reported good health.

In order to explore the difference in thermal comfort of outdoor people under different sunshine, 2395 questionnaires were divided into the following three groups according to different sunshine sensitivity:

Group A, sunshine sensitivity is not strong. There were 678 questionnaires in group A, including 232 in autumn and 466 in winter (Group A was divided based on those who selected "− 1" for the question "Sunshine sensitivity" in the subjective questionnaire).

Group B, sunshine sensitivity is moderate. There were 732 questionnaires in group B, including 258 in autumn and 474 in winter (Group B was divided based on those who selected "0" for the question "Sunshine sensitivity" in the subjective questionnaire).

Group C, sunshine sensitivity is strong. There were 985 questionnaires in group C, including 534 in autumn and 451 in winter (Group V was divided based on those who selected "1" for the question "Sunshine sensitivity" in the subjective questionnaire).

### Neutral temperature

Neutral temperature is the temperature when the human body is neither cold nor hot, that is, the temperature when the thermal sensation vote is 0. [29] The data analysis in this study was conducted using a combination of the Universal Thermal Climate Index (UTCI) and subjective questionnaire data. The UTCI is a comprehensive index that takes into account various environmental parameters, including air temperature, radiant temperature, humidity, and wind speed. Therefore, the actual measured temperature and other environmental factors are inherently considered in our analysis through the use of the UTCI. In order to study the relationship between thermal sensation and microclimate environment, UTCI (Universal Thermal Climate Index) within each ± 0.5 °C interval was an integer, and the mean voting value of thermal sensation (MTSV) corresponding to each operating temperature interval was calculated. The linear regression equation between UTCI (universal thermal climate index) and UTCI (MTSV = aUTCI + B) was established. If MTSV = 0, the neutral temperature can be obtained.

### Ethics declarations

The institutional review board of Xi’an Eurasia University approved the study protocol before data collection. Informed consent was obtained for all survey questionnaire participants. All methods were carried out in accordance with relevant guidelines and regulations. The subjects of the experiments in this paper were clear about the purpose of the experiments before completing the questionnaire and all of them agreed to conduct the experiments.

### Consent to participate

All the authors will participate in the review and publication process.

## Result

### Thermal sensation voting (TSV)

**Figure 4:**
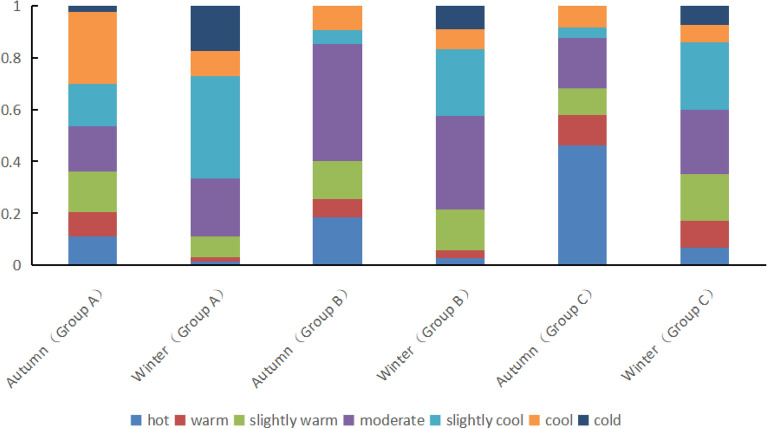
Outdoor thermal sensation voting of people under different sunshine sensitivity.

Figure 4 presents the thermal sensation voting chart of the crowd under different sunshine sensitivity.

Upon analyzing Fig. 4, it can be inferred that in autumn, 53.4% of people in group A felt hot (TSV ≥ 0), 85.3% of people in group B felt hot (TSV ≥ 0), and 87.7% of people in group C felt hot (TSV ≥ 0). Similarly, in winter, 33.5% of people in group A felt hot (TSV ≥ 0), 57.6% of people in group B felt hot (TSV ≥ 0), and 60.7% of people in group C felt hot (TSV ≥ 0).

A comprehensive analysis of Fig. 4 shows that the proportion of people feeling hot in group C (87.7% in autumn and 60.7% in winter) is much higher than that in group A (53.4% in autumn and 33.5% in winter). As outdoor sunshine sensitivity increases, the proportion of people feeling hot outdoors will also increase.

### Neutral temperature

The collected parameters, including humidity, temperature, wind speed, solar radiation, activity, and clothing index, were input into the Rayman software to obtain the corresponding UTCI value for each participant. The obtained Mean Thermal Sensation Vote (MTSV) and Universal Thermal Climate Index (UTCI) values of groups A, B, and C in autumn and winter were further analyzed through regression analysis using SPSS software, and a regression equation between MTSV and UTCI was established. For our regression analysis, we employed a linear regression model, taking into account the UTCI variable. The variables were selected based on their potential impact on thermal comfort. The regression was performed using SPSS (Statistical Package for the Social Sciences), And check and satisfy the assumptions of linearity, independence, homoscedasticity, and normality.

**Figure 5:**
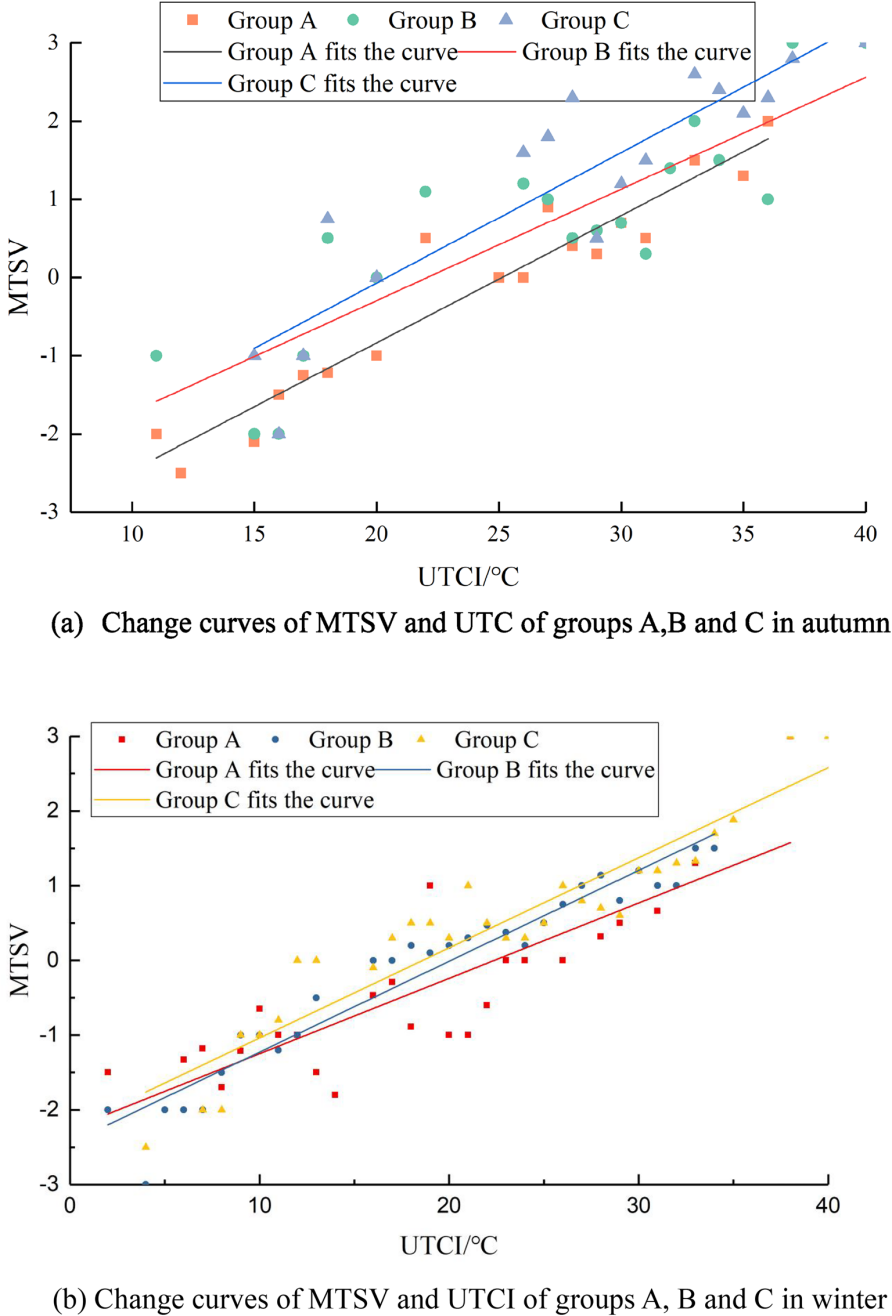
MTSV and UTC curves of groups A, B and C in different seasons.

Figure 5a displays the MTSV and UTCI values of groups A, B, and C in autumn, while Fig. 5b illustrates the MTSV and UTCI values of groups A, B, and C in winter. Meanwhile, Table 4 shows the outdoor neutral temperature of groups A, B, and C in autumn, and Table 5 shows the outdoor neutral temperature of groups A, B, and C in winter.

**Table 4:** A, B and C group's outdoor neutral temperature in autumn.

	Regression equation of UTCI and MTSV		Neutral temperature /°C
	Regression equation	R^2^	
Group A	MTSV = 0.165UTCI − 4.1345	0.9165	25.05
Group B	MTSV = 0.1429UTCI − 3.1543	0.7369	22.07
Group C	MTSV = 0.1671UTCI − 3.411	0.8069	20.41

**Table 5:** A, B and C groups' outdoor neutral temperature in winter.

	Regression equation of UTCI and MTSV		Neutral temperature /°C
	Regression equation	R^2^	
Group A	MTSV = 0.1008UTCI − 2.2569	0.7158	22.39
Group B	MTSV = 0.1215UTCI − 2.4411	0.9397	20.09
Group C	MTSV = 0.1206UTCI − 2.2438	0.8761	18.61

A comprehensive analysis of the data reveals that the neutral temperature of the crowd varies with the level of sunshine sensitivity. In autumn, group A experienced the weakest sunshine sensitivity, and the outdoor neutral temperature of group A was 25.05 °C. Conversely, group C experienced the most intense sunshine sensitivity, and the outdoor neutral temperature of group C was 20.41 °C. Therefore, in autumn, as sunshine sensitivity intensifies, the neutral temperature of the crowd decreases. Similarly, in winter, the outdoor neutral temperature of group A was 22.39 °C, that of group B was 20.09 °C, and that of group C was 18.61 °C, indicating that the crowd’s demand for outdoor temperature decreases with an increase in sunshine sensitivity.

### Thermal comfort vote (TCV)

**Figure 6:**
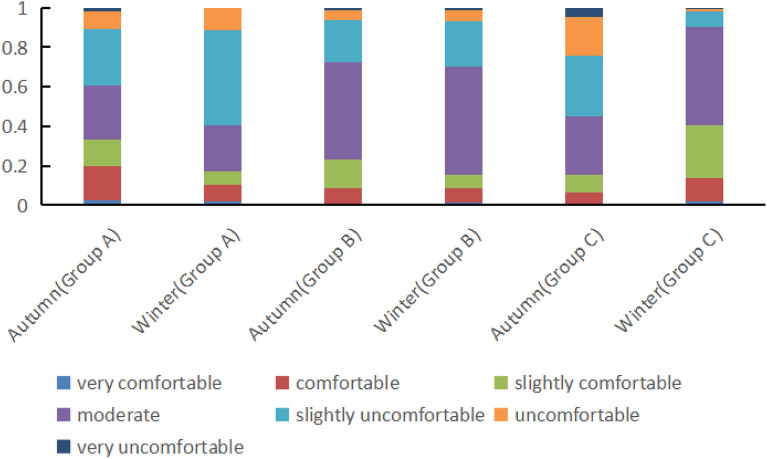
Outdoor thermal comfort voting of people under different sunshine sensitivity.

Figure 6 displays the voting chart of outdoor thermal comfort of people under different sunshine sensitivity.

According to the analysis of Fig. 6, in autumn, the proportion of people in group A feeling comfortable (TCV ≥ 0) was 60.9%, the proportion of people in group B feeling comfortable (TCV ≥ 0) was 72.2%, and the proportion of people in group C feeling comfortable (TCV ≥ 0) was 45.1%.

Similarly, in winter, the proportion of people in group A feeling comfortable (TCV ≥ 0) was 40.6%, the proportion of people in group B feeling comfortable (TCV ≥ 0) was 70.4%, and the proportion of people in group C feeling comfortable (TCV ≥ 0) was 90.5%.

A comprehensive analysis of Fig. 6 reveals that in autumn, comparing the TCV results of group A, B and C, it was found that the discomfort rate of group A was 39.1% (TCV < 0), group B was 27.8% (TCV < 0), and group C was 54.9% (TCV < 0). This result indicates that the population would feel uncomfortable in the outdoor area with strong sunshine sensitivity. However, the appropriate reduction of sunshine sensitivity can improve the comfort of the crowd. Conversely, in winter, insufficient sunshine sensitivity will reduce the thermal comfort of the crowd. With the strengthening of sunshine sensitivity, the thermal comfort of the crowd increases significantly, and the thermal comfort of group C reaches more than 90%.

### Expected temperature

In the outdoor environment, people's expectation of heat refers to their expectation of whether the outdoor temperature will increase, decrease, or remain unchanged. In the outdoor environment, people's expectation of heat indicates whether they anticipate the outdoor temperature to increase, decrease, or remain unchanged^23^. This expectation is described as "Prefer cooler conditions", "Prefer warmer conditions", or "Hope to remain unchanged". People's expectations of outdoor thermal comfort can directly reflect their satisfaction with the outdoor thermal environment at that time.Statistical analyses were conducted to quantify these expectations, with significance levels set at p < 0.05. When subjects "Prefer cooler conditions" or "Prefer warmer conditions", it indicates that they are not satisfied with the outdoor thermal environment, while "Hope to remain unchanged" indicates satisfaction.

**Figure 7:**
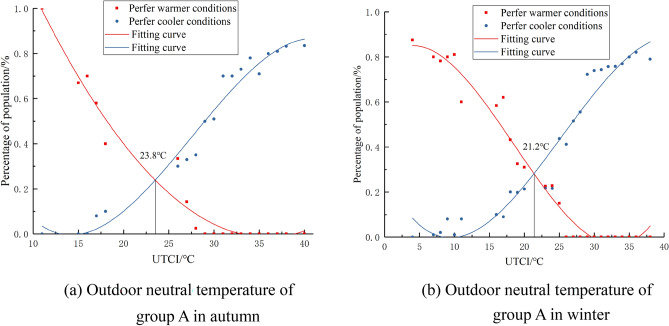
Fitting curve of the outdoor neutral temperature of group A.

**Figure 8:**
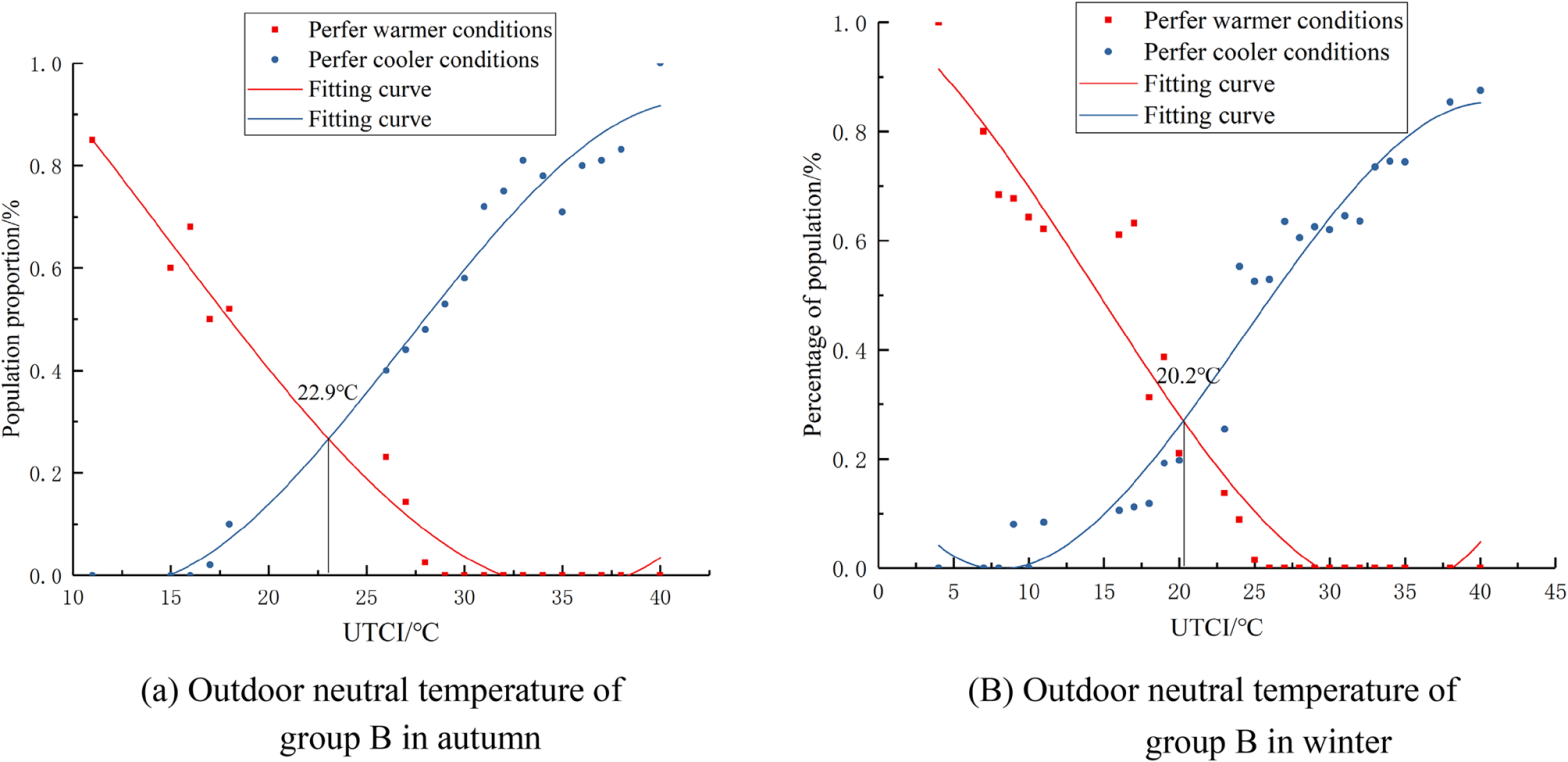
Fitting curve of the outdoor neutral temperature of group B.

**Figure 9:**
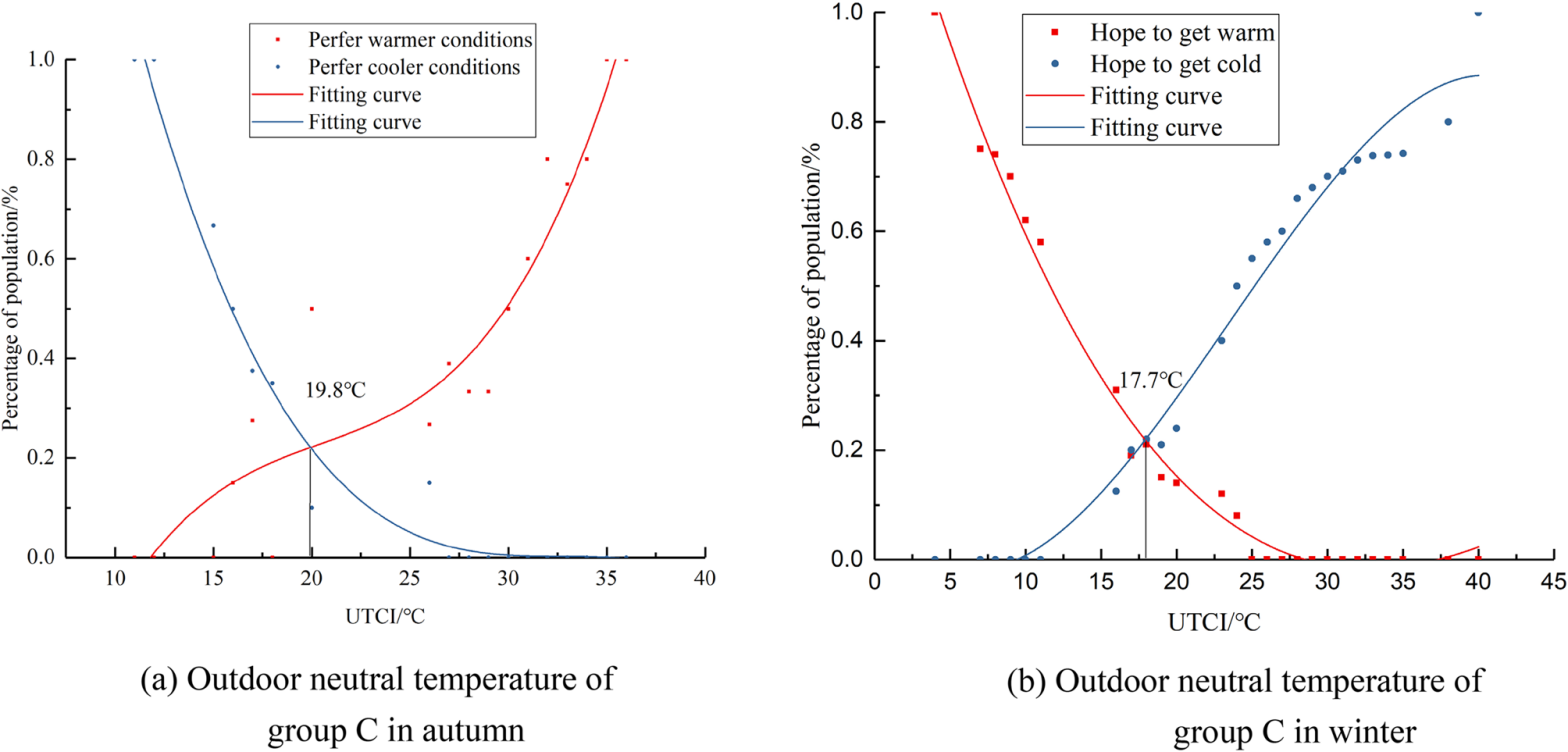
Fitting curve of the outdoor neutral temperature of group C.

Expected temperature represents the state where people do not want to be hotter or colder, this paper calculates the psychological expected temperature of groups A, B, and C The “expected temperature” denotes the thermal state where individuals neither prefer warmth nor coldness. In this study, we quantitatively assessed the psychological expected temperature across groups A, B, and C. We segmented outdoor temperatures based on 1 °C intervals of the UTCI. For each segment, we calculated and statistically analyzed the proportions of participants expressing a desire for cooler or warmer conditions, ensuring that the differences were statistically significant (p < 0.05), and the corresponding fitted curves were generated as shown in Figs. 7, 8, 9, and the intersection of the two desired curves indicated the desired temperature, as shown in Tables 6 and 7.

**Table 6:** Expected outdoor temperature of groups A,B and C in autumn.

		Fitting equation of percentage of people expected to cooler conditions and expected to warmer conditions		Expected temperature /°C
		Fitting equation	R^2^	
Group A	Cooler	y = 8*10^−6^x^3^ + 0.001x^2^ − 0.1031x + 1.9858	0.96	23.8
	Warmer	y = − 8*10^−5^x^3^ + 0.0066x^2^ − 0.1379x + 0.8504	0.97	
Group B	Cooler	y = 6*10^−5^x^3^ − 0.0033x^2^ + 0.0085x + 1.0601	0.98	22.9
	Warmer	y = − 1*10^−4^x^3^ + 0.0091x^2^ − 0.1814x + 1.0663	0.98	
Group C	Cooler	y = − 1*10^−4^x^3^ + 0.0103x^2^ − 0.3374x + 3.6779	0.98	19.8
	Warmer	y = 2*10^−4^x^3^ − 0.0101x^2^ + 0.2206x − 1.4679	0.89

**Table 7:** Expected outdoor temperature of groups A, B and C in winter.

		Fitting equation of percentage of people expected to cooler conditions and expected to warmer conditions		Expected temperature /°C
		Fitting equation	R^2^	
Group A	cooler	y = 7*10^−5^x^3^ − 0.004x^2^ + 0.0292x + 0.793	0.97	21.2
	warmer	y = − 6*10^−5^x^3^ + 0.0042x^2^ − 0.065x + 0.2798	0.95	
Group B	cooler	y = 4*10^−5^x^3^ − 0.0016x^2^ − 0.0191x + 1.0146	0.94	20.2
	warmer	y = − 5*10^-5^x^3^ + 0.0036x^2^ − 0.0488x + 0.1818	0.96	
Group C	cooler	y = − 1*10^-5^x^3^ + 0.0022x^2^ − 0.1011x + 1.3963	0.99	17.7
	warmer	y = − 5*10^−5^x^3^ + 0.0033 × 2 − 0.0368x + 0.0953	0.98	

From the analysis of Figs. 7, 8, 9 and Tables 6, 7, it can be observed that people's expected outdoor temperature changes with the degree of outdoor sunshine sensitivity An analysis of Figs. 7, 8, 9 and Tables 6, 7 reveals a statistically significant relationship between individuals' expected outdoor temperatures and varying sunshine sensitivity. T-tests and ANOVA were employed to determine the significance of these observed differences, with results indicating p-values less than 0.05. This paper draws the same conclusion for subjects in autumn and winter: the higher the outdoor sunshine sensitivity, the lower the expected temperature of people outdoors. Our findings for both autumn and winter indicate that as outdoor sunshine sensitivity increases, people's expected outdoor temperature decreases.

## Discussion

### Hypothesis validation

In this study, it was hypothesized that there are subjective differences in the thermal sensation of people outdoors under different sunlight intensities. Section “Thermal sensation voting (TSV)” of this paper provides evidence to support this hypothesis. Speak et al.^24^ conducted research in Bolzano, Northern Italy, and discovered that the Universal Thermal Climate Index (UTCI) of people exposed to sunlight was significantly higher than those in the shade. The foreheads of people exposed to the sun were 1.5 °C warmer than those in the shade. Similarly, Liu et al.^25^ conducted research at Qingdao University of Technology and found that the skin temperature of the human body increased significantly after sunshine sensitivity. These studies support the hypothesis of this paper. In the research site of this study, it was concluded that in autumn, the neutral temperature of people outdoors with low sunshine sensitivity was 25.05 °C, while the neutral temperature of people outdoors with moderate and strong sunshine sensitivity was 22.07 °C and 20.41 °C, respectively. In winter, the neutral temperature of people outdoors with low sunshine sensitivity was 22.39 °C, while the neutral temperature of people outdoors with moderate and strong sunshine sensitivity was 20.09 °C and 18.61 °C, respectively.

Another key hypothesis of this study is that there are subjective differences in the thermal comfort of outdoor individuals at different sunlight intensities. The findings in Section “Neutral temperature” support this hypothesis. Dong Wei et al.^26^ conducted a study on outdoor thermal comfort in Chengdu and found that people tend to prefer areas with stronger sunlight in winter, and higher levels of sunshine sensitivity enhance thermal comfort in winter. Hong Jin et al.^27^ observed in their study in Harbin that air temperature and solar exposure have an impact on outdoor comfort. In winter, as sunshine sensitivity increases, so does outdoor thermal comfort. The research conducted by these scholars reinforces the hypothesis proposed in this article. It can be concluded that on the research site of this paper, the expected temperature of individuals with low sunshine sensitivity is 23.8 °C in autumn, 22.9 °C for those with moderate sunshine sensitivity, and 19.8 °C for those with strong sunshine sensitivity. In winter, the expected temperature for individuals with low sunshine sensitivity is 21.2 °C, 20.02 °C for those with moderate sunshine sensitivity, and 17.7 °C for those with strong sunshine sensitivity.

This study aimed to discern the subjective differences in thermal sensations experienced by individuals outdoors under varying sunlight intensities. Notably, our results emphasize the significant role of sunshine sensitivity in influencing thermal comfort, especially in colder seasons. For instance, our data from the research site indicates that as sunshine sensitivity intensifies, the expected temperature for individuals decreases, both in autumn and winter. This observation underscores the intricate relationship between sunshine sensitivity and outdoor thermal comfort, especially in colder regions. The variations in neutral and expected temperatures across different sunlight intensities suggest that sunlight plays a pivotal role in shaping individuals' thermal perceptions.

### Innovation

The paper presents an innovative approach of classifying the outdoor crowd on campus based on different intensities of sunshine sensitivity, and investigates the impact of sunshine sensitivity on the thermal comfort of outdoor people. While many studies have examined the influence of sunshine sensitivity on outdoor thermal comfort, their analysis has been limited to identifying sunshine sensitivity as an important parameter for the assessment of thermal comfort. Through a comparative study of college students in Xi'an, the paper demonstrates that both neutral temperature and expected temperature of people exposed to the sun decrease. Interestingly, in winter, increasing sunshine sensitivity can improve outdoor thermal comfort of people.

Our findings underscore the intricate relationship between sunshine sensitivity and outdoor thermal comfort, especially in colder regions. The variations in neutral and expected temperatures across different sunlight intensities suggest that sunlight plays a pivotal role in shaping individuals' thermal perceptions. This has implications for urban planning and design, especially in university campuses where outdoor spaces are frequented by students. Future research could delve deeper into understanding the physiological responses to varied sunshine sensitivity and how they interplay with subjective thermal comfort perceptions.

### Limitations of the study

The research in this paper is mainly aimed at college students in Xi'an, and the subjects are all college students, so there is a lack of children and elderly samples. As a result, the conclusions of the article are not necessarily applicable to children and the elderly. Since the conclusion of this paper is based on a large sample, when conducting research in other areas, experiments should be carried out according to the research region and the population of subjects, and the results of this paper cannot be directly applied.

While our study primarily relied on subjective perceptions of the respondents, we acknowledge the importance of physiological measurements, such as skin temperature, in understanding the sensation of heat. Such measurements offer a direct insight into participants' thermal experiences. The absence of these physiological measurements in our study is a limitation. We also recognize that while environmental conditions provide context, they don't directly measure individual heat sensations. In future studies, incorporating both environmental and physiological measurements would provide a more comprehensive understanding of outdoor thermal comfort.

## Conclusion

In conclusion, this study investigated the thermal comfort of outdoor people on university campuses in cold areas under different sunshine sensitivitys. Our findings show that there are in the thermal comfort of outdoor people under different sunlight intensities. Specifically, in autumn, neutral temperatures under low, moderate, and strong sunshine sensitivity were 25.05 °C, 22.07 °C, and 20.41 °C respectively. In winter, these temperatures were 22.39 °C, 20.09 °C, and 18.61 °C. Expected temperatures in autumn were 23.8 °C, 22.9 °C, and 19.8 °C under low, moderate, and strong sunshine sensitivity, and in winter, these temperatures were 21.2 °C, 20.2 °C, and 17.7 °C.

This study contributes to the existing literature by classifying the outdoor crowd on campus according to different intensities of sunshine sensitivity and exploring the influence of sunshine sensitivity on the thermal comfort of the outdoor crowd. Our research expands upon previous studies that have only acknowledged the importance of sunshine sensitivity in outdoor thermal comfort evaluation. The results of this study can provide valuable insights for the design and planning of outdoor spaces in cold areas, and can help to optimize the outdoor thermal comfort of college students.

Drawing from 2395 valid questionnaires, this study integrates questionnaire surveys with field measurements to discern the variances in outdoor thermal comfort experienced by individuals under different sunlight perceptions. By juxtaposing our findings with similar research conducted in diverse study areas, we aimed to elucidate the influence of sunlight perception on human thermal comfort in outdoor public spaces. The salient conclusions derived from our research are:

Impact of Sunlight Perception on Thermal Comfort: In the outdoor environments of universities located in cold regions, varying sunlight perceptions lead to noticeable differences in thermal comfort. As individuals perceive increased sunlight, a larger proportion report feeling hot. Specifically, in autumn, the neutral temperatures are 25.05 °C for those with low sunlight perception, 22.07 °C for moderate, and 20.41 °C for strong sunlight perception. In winter, these temperatures are 22.39 °C, 20.09 °C, and 18.61 °C, respectively. Additionally, expected temperatures in autumn are 23.8 °C, 22.9 °C, and 19.8 °C for low, moderate, and strong sunlight perceptions, while in winter, they are 21.2 °C, 20.02 °C, and 17.7 °C, respectively.

Seasonal Adjustments for Optimal Comfort: In autumn, increasing shade in outdoor university spaces can enhance thermal comfort. However, in winter, increasing sunshine sensitivity proves beneficial for outdoor thermal comfort.

Implications for Planning: This research delves into the thermal comfort of individuals in outdoor university spaces in cold regions, contingent on their sunlight sensitivities. The findings underscore that adjusting sunshine sensitivity, based on the season, can optimize outdoor comfort. Such insights are not only pivotal for planning university infrastructures in cold regions but also serve as valuable references for designing other outdoor spaces within similar climatic zones.

## Data Availability

Data will be provided on request. If anyone would like data from this study, please contact the corresponding author of this article.

## References

[1] Yuting LIU (2018). Research on External Space Comfort of High-Density University Campus in Lingnan Region Based on Microclimate.

[2] Ma X, Tian Y, Du M, Hong B, Lin B (2021). How to design comfortable open spaces for the elderly? Implications of their thermal perceptions in an urban park. Sci. Total Environ..

[3] Li J, Liu N (2020). The perception, optimization strategies and prospects of outdoor thermal comfort in China: A review. Build. Environ..

[4] Xu X, Sun S, Liu W, García EH, He L, Cai Q, Zhu J (2017). The cooling and energy saving effect of landscape design parameters of urban park in summer: A case of Beijing, China. Energy Build..

[5] Chen X, Xue P, Liu L, Gao L, Liu J (2018). Outdoor thermal comfort and adaptation in severe cold area: A longitudinal survey in Harbin, China. Build. Environ..

[6] American National Standards Institute. (2004). Thermal environmental conditions for human occupancy (Vol. 55, No. 2004). American Society of Heating, Refrigerating and Air-Conditioning Engineers.

[7] Gonzalez RR, Nishi Y, Gagge AP (1974). Experimental evaluation of standard effective temperature a new biometeorological index of man's thermal discomfort. Int. J. Biometeorol..

[8] Mayer H, Höppe P (1987). Thermal comfort of man in different urban environments. Theoret. Appl. Climatol..

[9] Liu S, Xie Y, Zhu Y, Lin B, Cao B, Wong NH, Ignatius M (2022). Comparative analysis on indoor and outdoor thermal comfort in transitional seasons and summer based on multiple databases: Lessons learnt from the outdoors. Sci. Total Environ..

[10] Jendritzky G, de Dear R, Havenith G (2012). UTCI—Why another thermal index?. Int. J. Biometeorol..

[11] Nagano K, Horikoshi T (2011). Development of outdoor thermal index indicating universal and separate effects on human thermal comfort. Int. J. Biometeorol..

[12] Nagano K, Horikoshi T (2011). New index indicating the universal and separate effects on human comfort under outdoor and non-uniform thermal conditions. Energy Build..

[13] Kurazumi Y, Tsuchikawa T, Kondo E, Horikoshi T, Matsubara N (2010). Conduction-corrected modified effective temperature as the indices of combined and separate effect of environmental factors on sensational temperature. Energy Build..

[14] Kenny NA, Warland JS, Brown RD, Gillespie TG (2009). Part A: Assessing the performance of the COMFA outdoor thermal comfort model on subjects performing physical activity. Int. J. Biometeorol..

[15] McQuinston F, Spitler J (1992). Cooling and Heating Load Calculation Manual.

[16] Höppe P (1999). The physiological equivalent temperature–a universal index for the biometeorological assessment of the thermal environment. Int. J. Biometeorol..

[17] Blazejczyk K, Epstein Y, Jendritzky G, Staiger H, Tinz B (2012). Comparison of UTCI to selected thermal indices. Int. J. Biometeorol..

[18] Fiala D, Havenith G, Bröde P, Kampmann B, Jendritzky G (2012). UTCI-Fiala multi-node model of human heat transfer and temperature regulation. Int. J. Biometeorol..

[19] Nikolopoulou M, Lykoudis S (2006). Thermal comfort in outdoor urban spaces: Analysis across different European countries. Build. Environ..

[20] Andrade H, Alcoforado MJ, Oliveira S (2011). Perception of temperature and wind by users of public outdoor spaces: Relationships with weather parameters and personal characteristics. Int. J. Biometeorol..

[21] Lai D, Guo D, Hou Y, Lin C, Chen Q (2014). Studies of outdoor thermal comfort in northern China. Build. Environ..

[22] Lin TP, De Dear R, Hwang RL (2011). Effect of thermal adaptation on seasonal outdoor thermal comfort. Int. J. Climatol..

[23] Zheng WX (2017). Effects of Regional and Seasonal Climate Change on Human Thermal Adaptation and its Application.

[24] Speak A, Montagnani L, Wellstein C, Zerbe S (2021). Forehead temperatures as an indicator of outdoor thermal comfort and the influence of tree shade. Urban Climate.

[25] Liu G, Wang Z, Li C, Hu S, Chen X, Liang P (2020). Heat exchange character and thermal comfort of young people in the building with solar radiation in winter. Build. Environ..

[26] Wei D, Yang L, Bao Z, Lu Y, Yang H (2022). Variations in outdoor thermal comfort in an urban park in the hot-summer and cold-winter region of China. Sustain. Cities Soc..

[27] Jin H, Liu S, Kang J (2020). Gender differences in thermal comfort on pedestrian streets in cold and transitional seasons in severe cold regions in China. Build. Environ..

